# PACES: prediction of N4-acetylcytidine (ac4C) modification sites in mRNA

**DOI:** 10.1038/s41598-019-47594-7

**Published:** 2019-07-31

**Authors:** Wanqing Zhao, Yiran Zhou, Qinghua Cui, Yuan Zhou

**Affiliations:** 1 Department of Biomedical Informatics, Department of Physiology and Pathophysiology, Center for Noncoding RNA Medicine, MOE Key Lab of Cardiovascular Sciences, School of Basic Medical Sciences, Peking University, 38 Xueyuan Rd, Beijing, 100191 China; 2 Center of Bioinformatics, Key Laboratory for Neuro-Information of Ministry of Education, School of Life Science and Technology, University of Electronic Science and Technology of China, Chengdu, 610054 China

**Keywords:** Sequence annotation, Machine learning

## Abstract

N4-acetylcytidine (ac4C) is a highly conserved RNA modification and is the first acetylation event described in mRNA. ac4C in mRNA has been demonstrated to be involved in the regulation of mRNA stability, processing and translation, but the exact means by which ac4C works remain unclear. In addition, ac4C is widely distributed within the human transcriptome at physiologically relevant levels and so far only a small fraction of modified sequences have been detected by experiments. In this study, we developed a predictor of ac4C sites in human mRNA named PACES to help mining possible modified motifs. PACES combines two random forest classifiers, position-specific dinucleotide sequence profile and K-nucleotide frequencies. With genomic sequences as input, PACES gives possible modified sequences based on the training model. PACES is freely available at http://www.rnanut.net/paces/.

## Introduction

With recent emerging studies in epitranscriptome, over 160 different modifications have been identified in RNA molecules^1^. Among all the modifications that have regulatory potentials, N4-acetylcytidine (ac4C) which occurs on cytidine is conserved in all domains of life^2^ and is the sole acetylation event that has been described in eukaryotic RNA^3^. ac4C was initially detected in the bacterial tRNA^met ^^4^ and then was subsequently detected in other tRNA and rRNA^5^. In a variety of human cells, recent studies have detected ac4C in poly(A) RNA on a level with 5′ 7-methylguanosine (m7G) cap^6^, which suggests that ac4C is abundant in mRNA and it has important regulatory functions.

More recently, Daniel Arango *et al*. established a role for ac4C in the regulation of mRNA translation and explored the mechanism by which ac4C promotes translation efficiency^7^. Through analysis of mRNA half-lives, they found the acetylation level was positively correlated with the stability of target mRNA and ac4C enhanced translation when presented within wobble cytidine^7^. A similar mechanism has been found for prokaryotic tRNA^met^ acetylation, where ac4C promotes decoding fidelity in prokaryotes^8,9^. Besides, ac4C occurring in wobble site where mRNA/tRNA interactions allow for non-standard base-pairing may support tRNA recognition and promote interaction with cognate tRNAs^10^.

Utilizing high-throughput sequencing approach named acRIP-seq^7^, Daniel Arango *et al*. also depicted a transcriptome-wide map of ac4C. Overall, ac4C is enriched within coding sequences and cytidine is strongly enriched within wobble sites in acRIP-seq peaks. Indeed, analysis of specific motifs demonstrates that repeating CXX motifs (several obligate cytidines separated by two non-obligate nucleotides) are highly enriched in ac4C peaks. While the experiment results have revealed some characteristics of acetylated sequences, the basis by which some wobble cytidine codons are acetylated and others are not remains obscure. In this study, we establish an ac4C site predictor named PACES to help finding more acetylated mRNA sequences.

In previous work in our lab, there are already several predictors for modification sites established. SRAMP is a computational predictor that combines three random forest classifiers to accurately identify the mammalian N6-methyladenosine (m6A) sites^11^. RNAm5Cfinder is a web-server based on machine learning to predict 5-methylcytosine (m5C) sites in RNA^12^. NmSEER is also a predictor utilizing random forest to predict 2′-O-methylation (Nm) sites in Hela cells and HEK293 cells^13^. Encouraged by the success of using machine learning methods to predict modification sites, we establish PACES based on RNA sequence features and random forest machine learning. Different from the three predictors mentioned above, PACES makes predictions at the motif level. PACES combines position-specific dinucleotide sequence profile (PSDSP) and K-nucleotide frequencies (KNF) to extract sequence features. PACES shows promising performance in cross-validation tests and independent benchmarking tests and we believe it can help subsequent researches on ac4C regulation mechanisms.

## Results

### Predictor establishment

The dataset we use is extracted from the recently published article^7^. The experiment identified 2135 ac4C peaks and provided their gene ID and the position of peak. We extracted positive samples and negative samples from these ac4C sequences using repeating CXX motifs for subsequent machine learning (Methods). After optimizing, we finally selected at least five continuous CXX repeats and the adjacent sequences as a sample motif to build a dataset in which the radio of positive to negative samples was near 10:1. These samples were divided into training set and test set by a ratio close to 2.5:1. The difference between positive samples and negative samples that may affect the acetylation of the sequence is unclear. The single nucleotide, dinucleotide or k-nucleotides at specific locations, nucleotides compositions and the physicochemical properties of nucleotides are all possible factors. Therefore, we tried various ways to extract the sequence features of samples to form the feature vectors as input of the random forest classifier.

We have tried six different methods for constructing sequence features (Methods), one-hot^14–16^, position-specific nucleotide sequence profile (PSNSP)^17^, position-specific dinucleotide sequence profile (PSDSP)^18^, K-nucleotide frequencies (KNF)^19,20^, K-spaced nucleotide pair frequencies (KSNPF)^21^ and pseudo K-tuple nucleotide composition (PseKNC)^22,23^. One-hot is binary encoding that it translates four nucleotides A, T, C or G into a binary number from 1 to 4. One-hot exactly depicts the nucleotide type at each position of the sample sequences. PSNSP translates the nucleotide at each position into the difference between the frequencies of this nucleotide appearing at this position in the positive and negative sample sets respectively. It depicts the profile of a nucleotide at each position containing the information of the whole dataset. PSDSP is similar to PSNSP that it depicts the frequency difference for dinucleotides at every position. KNF calculates the frequencies of all possible k-nucleotides composition and depicts the sequence context. KSNPF calculates the frequencies of all possible nucleotide pair that separated by k arbitrary nucleotides and also depicts the sequence context. PseKNC combines the occurrence frequencies of all possible dinucleotide or trinucleotide composition and their certain physicochemical properties such as base stacking and protein-induced deformability.

**Figure 1:**
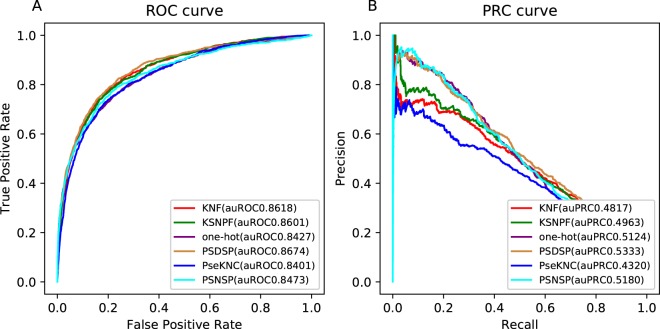
The performance of six classifiers in cross-validation. (**A**) ROC curve. (**B**) Precision-recall curve.

For every encoding method, after translating the training sample sequences into numeric feature vectors, we trained the random forest classifier with the matrix composed of these vectors. We assessed the performance of these classifiers with their false positive rate (FPR), true positive rate (TPR) and area under ROC curve (auROC) in 5-fold cross-validation test^24,25^. When focusing on the auROC, although the difference is small, PSDSP is obviously superior (auROC = 0.8674). All the six classifiers perform well according to auROC (Fig. 1), indicating that there is some information carried by sequence that can make predictor distinguish acetylated motifs from non-acetylated samples.

### Parameter optimization and independent testing

The performance of the classifiers may be affected by several parameters and in the process of training classifier, we focused on the window size W and the n_estimators (tree number) N. For every encoding approach, we optimized the two parameters through 5-fold cross-validation tests (Method). Generally, the auROC gradually converged after the window size grew over 100 nucleotides (Supplementary Fig. S1). Based on the performance of all the six approaches, we chose the optimized window size at 126 for one-hot, 144 for PSNSP, 138 for PSDSP, 150 for KNF, 144 for KSNPF and 138 for PseKNC encodings, respectively. Similarly, the n_estimators is preliminarily optimized to 800. Other parameters make little difference on the performance and we leave them as the default.

**Figure 2:**
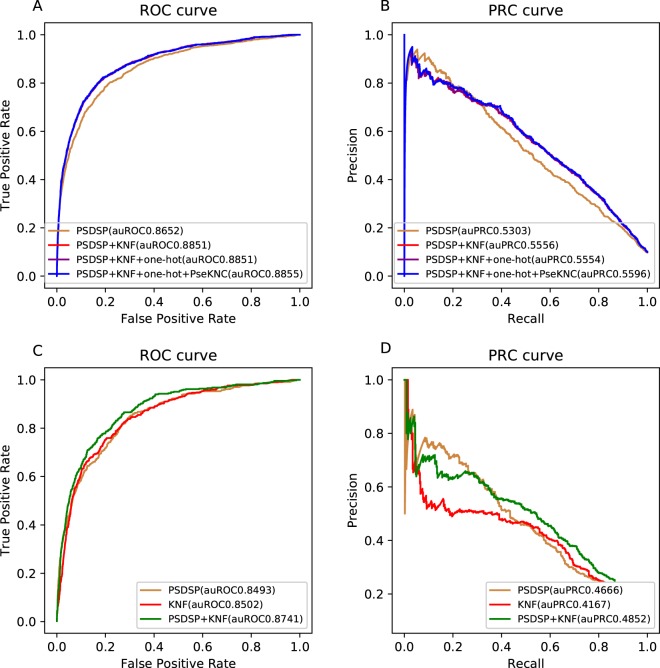
The performance of different classifiers and their combinations. (**A**) ROC curve showing the performance of different combinations of classifiers in cross-validation. (**B**) Precision-recall curve showing the performance of different combinations of classifiers in cross-validation. (**C**) ROC curve showing the performance of different combinations of classifiers and two single classifiers in independent test. (**D**) Precision-recall curve showing the performance of different combinations of classifiers and two single classifiers in independent test.

After optimizing every classifier, we combined the prediction results of them linearly by weighted sum schema to create a meta-classifier that would perform better than any single approach. We still used 5-fold cross-validation tests to find better classifiers combination and better classifier weights. In single classifier, PSDSP do the best (auROC = 0.8674), followed by KNF (auROC = 0.8618) and PseKNC (auROC = 0.8401) (Fig. 1A). We combined these single classifiers in order of their auROC value until the performance of the combination classifier in 5-fold cross-validation no longer got better. It shows that when combined with KNF (auROC = 0.8851), the meta-classifier performs better than the single PSDSP classifier, but the continued addition of one-hot and PseKNC don’t make the classifier significantly improved (auROC = 0.8855, Fig. 2A). Ultimately, the meta-predictor was established by combing random forest classifiers trained with PSDSP and KNF encodings, respectively.

To reasonably evaluate this combination classifier, we tested it on the independent testing dataset and compared it with the single PSDSP and KNF classifier (Fig. 2C). The result from the independent test was consistent with that from the cross-validation test. The combination classifier whose auROC reaches 0.8741 is significantly better than the two single classifiers (auROC = 0.8493 and 0.8502, respectively). And it also shows good performance based on another metric, area under precision-recall curve in both cross-validation test and independent test. The precision-recall curve is sensitive to the distribution of the positive and negative samples in the dataset. Both the area under the precision-recall curve (auPRC = 0.4852, Fig. 2D) and the BEP (break-event point) of the combination classifier are bigger than those of the other two classifiers (0.4666 and 0.4167, respectively). Therefore, we use the combination classifier as the predictor.

### The PACES server

**Figure 3:**
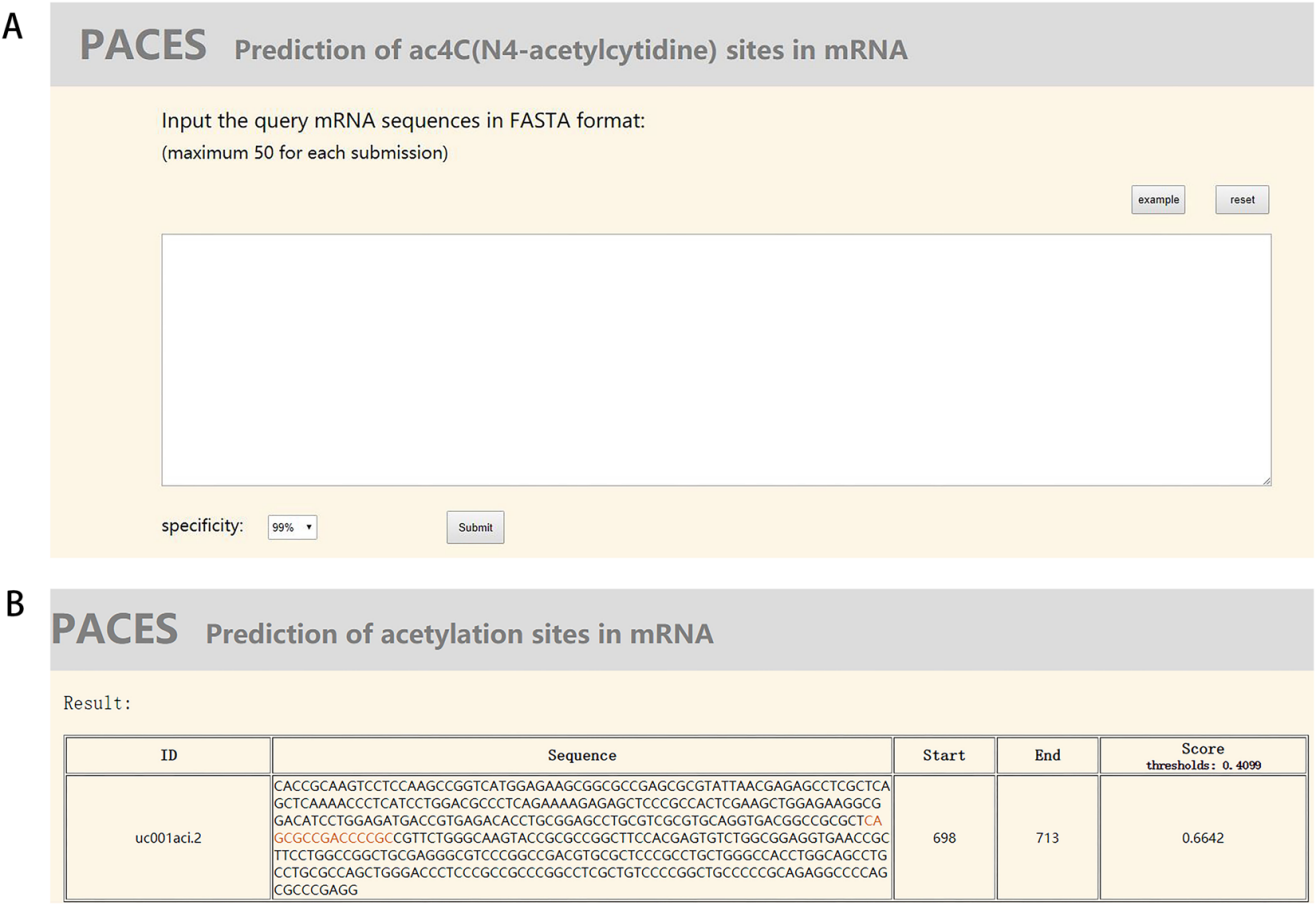
The PACES server. (**A**) The prediction webpage of PACES. (**B**) The result webpage of PACES.

To further facilitate the community, we built an online server PACES. PACES is a user-friendly web server allowing users to query for the possible acetylation motif of the sequence they provide. The prediction webpage of PACES is shown in Fig. 3A. Users should input the query RNA or cDNA sequences and select the specificity they want. The submission allows maximum 50 sequences at a time and the specificity what decides the threshold of the classifier have four alternatives provided to users: 99%, 95%, 90% and 85%. The default option of the specificity is 99%. After the query sequence is submitted, depending on the quantity of the sequences submitted by user, the processing can take a few seconds. Then the user will be redirected to the result webpage where only query sequences containing possible acetylation motifs are shown. Fig. 3B is a screenshot of the result webpage using the example sequence (UCSC ID: uc001aci.2) as the input. All the possible acetylated sequences in the query sequences and their ID are listed. If there are overlapped motifs (more than five continuous CXX repeats), the prediction score is calculated and compared for every five consecutive CXX motif among the overlapped motifs and only the CXX motif with the highest score will be shown. The possible acetylation motifs are marked red in the sequences and their starting and ending sites are also given. In addition, the score given by the predictor for each shown sequence is displayed for comparison.

## Discussion

Acetylation of mRNA appears to play an important role in the stability and translation of mRNA and the mechanism of acetylation and the accurate site of acetylation event remain unclear. The PACES server we have presented above is a sequence-based predictor for acetylation site in mRNA. It is built on the acetylated dataset from an experiment and can predict the query sequences whether have possible acetylation motifs. The predictor has shown promising predictive performance with auROC reaching 0.8741 on independent test.

Nonetheless, there are still some limitations. Firstly, PACES can only predict the motif where acetylation is likely to occur, not the exact location of acetylation. This is due to the limited resolution of high-throughput experiments to detect ac4C acetylation, from which only 100~300 nt acetylation peaks were obtained. The overrepresented CXX motifs near the summit of acetylation peak were selected as the ac4C acetylation motifs, but there is no guarantee that the acetylation should occur on this motif for every peak (for example, there could be multiple ac4C sites in one peak). Second, the repeating CXX motif is still ambiguous and the exact form of the ac4C acetylation, like the DRACH motif for m6A, needs further experimental investigation. Third, PACES cannot perform cross-tissue or cross-species prediction due to the limitation of available data. Therefore, to improve the accuracy of the predictor, more high-resolution experimental measurement is obligate.

In addition, when training the random forest classifier, we payed attention to the feature importance. In PSDSP classifier, four nucleotide positions at the closest proximity to the CXX motif rank the top four in the feature importance ranking, indicating local sequence preference does influence the selection of acetylation motif. But their importance score is not significantly different from other features, indicating the distal nucleotide positions may also contribute to the acetylation specificity. And in KNF classifier, in the 336 features, the top five are the frequencies of k-nucleotide containing GG (GG, GGG, GGGG, GGAG, GGA), suggesting a G-rich context favorable for ac4C acetylation. Whether this result truly reflect the nature of ac4C motif selection requires further experimental study.

## Methods

### Datasets

Both positive and negative samples were extracted from 2134 genes provided by previously published high-throughput dataset^7^. We changed the number of repetitions of CXX from 2 to 9 to see the distribution of different repeating CXX motifs within and outside of the acetylation peak. Simple repeating motifs like CXXCXX could be very frequently observed in transcriptome, but we found five consecutive CXX motif occurred in 1629 peaks and there were 15198 five consecutive CXX motifs found out of the peak, resulting an acceptable positive-to-negative ratio (1:10). Therefore, we chose at least five times repeating CXX motifs and their neighboring sequences as the criterion for collecting samples. We took one motif from each peak, and if the peak had more than one motif, then the motif closest to the summit was selected. We used motifs within the peaks as positive samples and those outside of the peaks as negative samples. Finally, these samples were divided into training set and test set. There were 1160 positive samples, 10855 negative samples in the training set and 469 positive samples, 4343 negative samples in the test set.

### Feature encoding

We tried six feature encoding approaches, one-hot, PSNSP, PSDSP, KNF, KSNPF, PseKNC. As for one-hot, we translated A, T, G, C into binary vectors (0,0,0,1), (0,0,1,0), (0,1,0,0), (1,0,0,0).

PSNSP (position-specific nucleotide sequence profile) depicts the distribution of the four nucleotides at every site in positive and negative datasets:

1 $${f}(i{,}n)=\frac{{{N}}^{+}(i{,}n)}{{{N}}_{p}}-\frac{{{N}}^{-}(i{,}n)}{{{N}}_{{n}}}$$

where *i* is the *i*-th site of the sequence and *n* is the nucleotide type at this site. *N*^+^(*i*, *n*) and *N*^−^(*i*, *n*) represent the counts of nucleotide *n* occurring at *i*-th site in the positive samples and negative samples respectively. *N*_*p*_ and *N*_*n*_ represent the number of positive samples and negative samples. When encoding, we translated nucleotide at every site into the value of *f* (*i*, *n*).

PSDSP (position-specific dinucleotide sequence profile) depicts the frequency difference for the dinucleotide at every site between positive and negative datasets. PSDSP is also calculated in a way similar to Eq. 1. The difference is *n* represents sixteen dinucleotides, i.e. AA, AT, AG, AC, TA, TT, TG, TC, GA, GT, GG, GC, CA, CT, CG, CC. By procedure, we translated nucleotides at *i*-th and (*i* + 1)-th positions into the value of *f* (*i*, *n*).

KNF (k-nucleotide frequencies) depicts the frequencies of all possible polynucleotide with k nucleotides occurring in the sequence. When k was set equal to 2, 3 and 4, the sequence is represented by the dinucleotide composition:

2 $$[f(AA)f(AT)\ldots f(AAA)f(AAT)\ldots f(AAAA)f(AAAT)\ldots ]$$

where *f* (*AA*) is the occurrence frequency of AA in the sequence. The frequencies of all the dinucleotide, trinucleotide and tetranucleotide were calculated and merged together as a vector. By procedure, we translated the sequence to a vector of length 336 (16 + 64 + 256 = 336).

KSNPF (k-spaced nucleotide pair frequencies) depicts the frequencies of sixteen nucleotides pair separated by k arbitrary nucleotides occurring in the sequence. We set k equal to 0, 1, 2, 3 and 4, then the sequence would be translated into:

3 $$[f(AA)\ldots f(AXA)\ldots f(AXXA)\ldots f(AXXXA)\ldots f(AXXXXA)\ldots ]$$

where X is an arbitrary nucleotide and AXA represents two adenosines separated by an arbitrary nucleotide. *f* (*AXA*) indicates the occurrence frequency of AXA in the sequence. The frequencies of all nucleotide pairs are calculated and the sequence is encoded to a vector of length 80 (16 × 5 = 80).

PseKNC (pseudo k-tuple nucleotide composition) combines the frequencies and physicochemical property of k-nucleotide composition. PseKNC has various parameters to generate different modes and the mathematical equations are complicated. Therefore, we used an available software package for PseKNC to generate PseKNC encoding feature vector for each sample^22^. As for the parameters for PseKNC, we preliminarily scanned the parameter combinations by grid search and found that there was only small difference in their performance (Supplementary Fig. S2). By comparison, we finally set the weight factor to 0.3, λ parameter to 1 and took all the 11 physicochemical properties of RNA dinucleotide into account.

### Random forest classifier training and optimization

The PACES predictor integrates two random forest classifiers and we used random forest package named RandomForestClassifier in sklearn package in Python^26,27^. When we used training dataset to do 5-fold cross-validation tests, we divided the training samples into five equal parts (each has 232 positive samples and 2171 negative samples). One of these five sets was taken as a test set in turn, and the other four were merged into a training set. After five trainings, the results of the five test sets were combined for performance assessment. Then, we optimized the two parameters window size and tree number n_estimators based on the performance of cross-validation. We changed the window size from 0 to 198 with the step size 6 and the tree number from 100 to 900 with the step size 100. Based on the performance of the six methods, we finally set the tree number at 800 and the window size at 126 for one-hot, 144 for PSNSP, 138 for PSDSP, 150 for KNF, 144 for KSNPF, and 138 for PseKNC.

## Supplementary information

Supplementary Figures

## Data Availability

The datasets used to build the model are available in our web-server PACES (http://rnanut.net/paces/dataset.zip).
