# Supplementary material for: PACES: prediction of N4-acetylcytidine (ac4C) modification sites in mRNA
Source: Sci Rep. 2019 Jul 31;9:11112. doi: 10.1038/s41598-019-47594-7 (PMC6668381; doi:10.1038/s41598-019-47594-7)
Supplement: Supplementary file 1 — Supplementary Figures [file 41598_2019_47594_MOESM1_ESM.docx]

***Supplementary information***

**PACES: prediction of N4-acetylcytidine (ac4C) modification sites in mRNA**

Wanqing Zhao^1^, Yiran Zhou^1^, Qinghua Cui^1,2,*^, Yuan Zhou^1,*^

^1^Department of Biomedical Informatics, Department of Physiology and Pathophysiology, Center for Noncoding RNA Medicine, MOE Key Lab of Cardiovascular Sciences, School of Basic Medical Sciences, Peking University, 38 Xueyuan Rd, Beijing 100191, China and ^2^Center of Bioinformatics, Key Laboratory for Neuro-Information of Ministry of Education, School of Life Science and Technology, University of Electronic Science and Technology of China, Chengdu 610054, China

^*^Corresponding authors:

Qinghua Cui, Tel: +86 10 82801585; Fax: +86 10 82801001; Email: [cuiqinghua@hsc.pku.edu.cn](mailto:cuiqinghua@hsc.pku.edu.cn). Yuan Zhou, Tel: +86 10 82801585; Fax: +86 10 82801001; Email: [zhouyuanbioinfo@hsc.pku.edu.cn](mailto:zhouyuanbioinfo@hsc.pku.edu.cn).


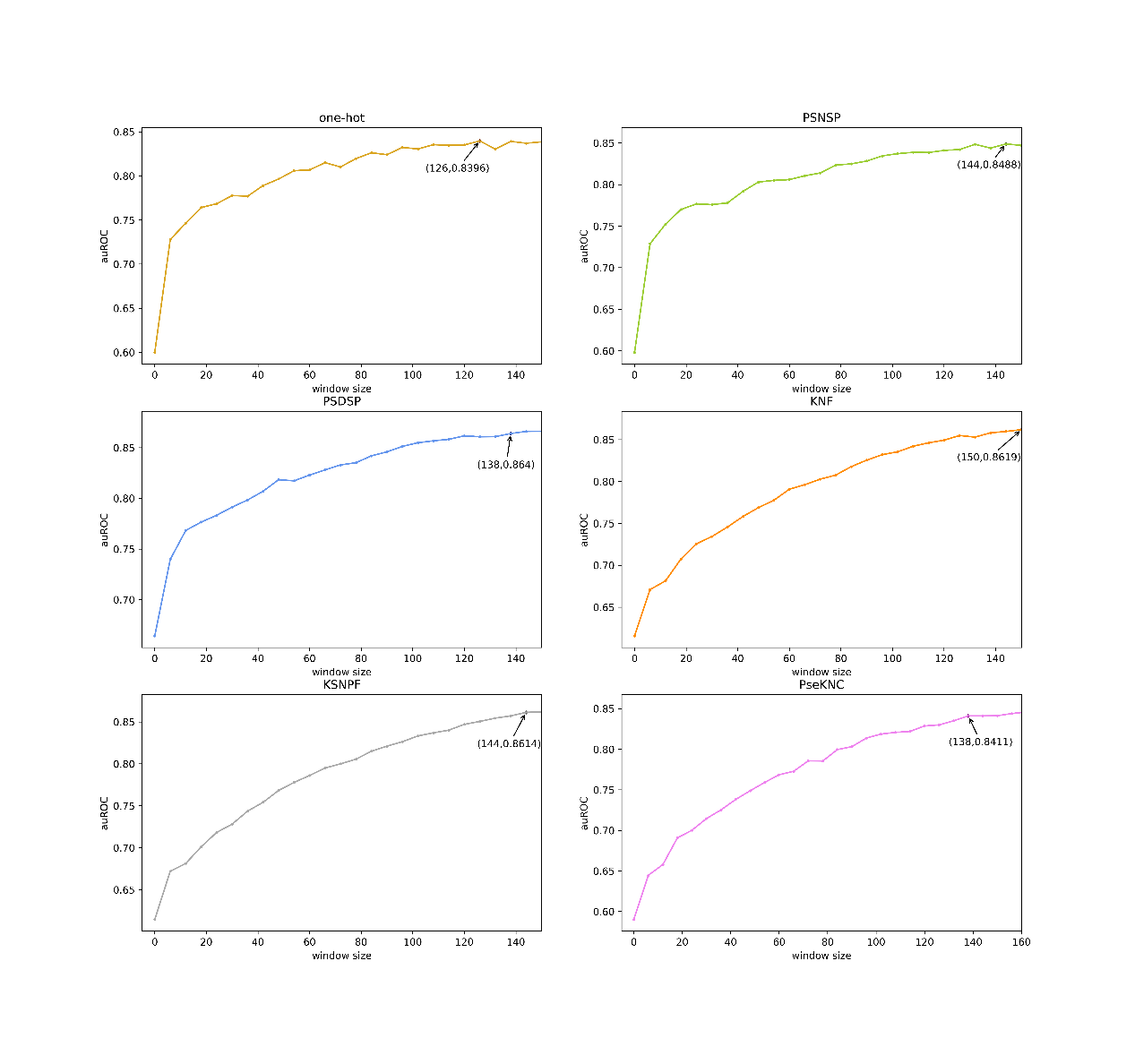


**Supplementary Figure S1. The growth of prediction performance with different window sizes for the six encodings.** The prediction performance is evaluated as the area under ROC curve (auROC) at the corresponding window size.


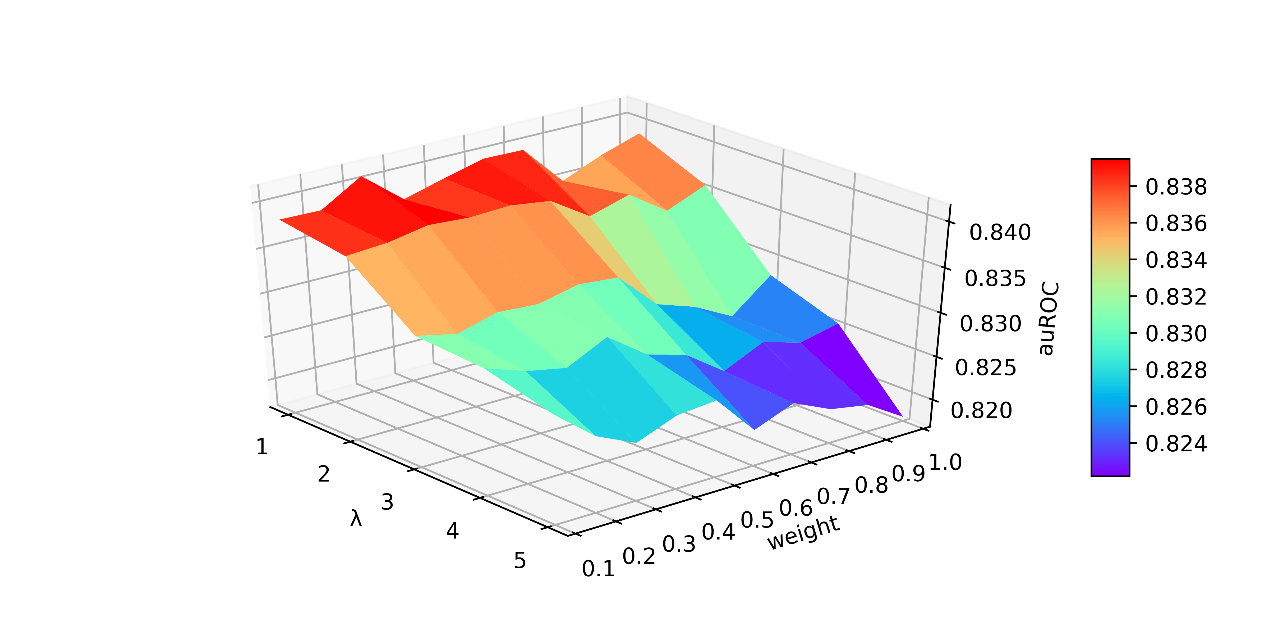


**Supplementary Figure S2. Surface plot showing the changing performance of PseKNC encoding with different parameters combinations.** By grid search, prediction performance with different combinations of two core parameters (i.e. λ and weight) for the PseKNC encoding are tested and compared. The prediction performance is evaluated as the area under ROC curve (auROC) at the corresponding λ and weight combination.
